# SPM—30 years and beyond

**DOI:** 10.1093/cercor/bhaf234

**Published:** 2025-09-10

**Authors:** Peter Zeidman, John Ashburner, Gareth Barnes, Olivia Kowalczyk, Christian Lambert, Vladimir Litvak, Thomas E Nichols, Thomas Parr, Tim M Tierney, Karl Friston

**Affiliations:** Functional Imaging Laboratory (FIL), Department of Imaging Neuroscience, University College London, 12 Queen Square, London WC1N 3AR, United Kingdom; Functional Imaging Laboratory (FIL), Department of Imaging Neuroscience, University College London, 12 Queen Square, London WC1N 3AR, United Kingdom; Functional Imaging Laboratory (FIL), Department of Imaging Neuroscience, University College London, 12 Queen Square, London WC1N 3AR, United Kingdom; Functional Imaging Laboratory (FIL), Department of Imaging Neuroscience, University College London, 12 Queen Square, London WC1N 3AR, United Kingdom; Functional Imaging Laboratory (FIL), Department of Imaging Neuroscience, University College London, 12 Queen Square, London WC1N 3AR, United Kingdom; Functional Imaging Laboratory (FIL), Department of Imaging Neuroscience, University College London, 12 Queen Square, London WC1N 3AR, United Kingdom; Big Data Institute, University of Oxford, Li Ka Shing Centre for Health Information and Discovery, Old Road Campus, Oxford OX3 7LF, United Kingdom; Nuffield Department of Clinical Neurosciences, University of Oxford, Level 6, West Wing, John Radcliffe Hospital, Oxford OX3 9DU, United Kingdom; Functional Imaging Laboratory (FIL), Department of Imaging Neuroscience, University College London, 12 Queen Square, London WC1N 3AR, United Kingdom; Functional Imaging Laboratory (FIL), Department of Imaging Neuroscience, University College London, 12 Queen Square, London WC1N 3AR, United Kingdom

**Keywords:** EEG, MEG, MRI, SPM, statistical parametric mapping

## Abstract

This paper marks the 30th anniversary of the Statistical Parametric Mapping (SPM) software and the journal *Cerebral Cortex*: two modest milestones that mark the inception of cognitive neuroscience. We take this opportunity to reflect on SPM, a generation after its introduction. Each of the authors of this paper—who represent a small selection of the many contributors to SPM—were asked to consider lessons learned, what has gone well, and where there is room for improvement in future development. We hope that this review of SPM—and its aspirations—will provide some context for current imaging neuroscience and foreground some potential directions for the future of the field.


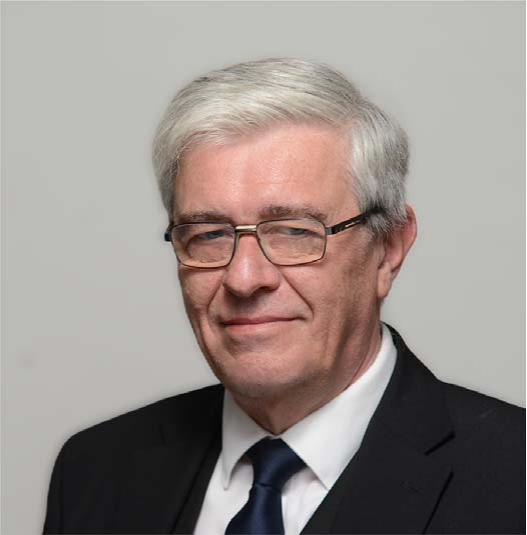


**Karl Friston** is a theoretical neuroscientist and authority on mathematical modelling. He invented statistical parametric mapping (SPM), voxel-based morphometry (VBM) and dynamic causal modelling (DCM). These contributions were motivated by schizophrenia research and theoretical studies of value-learning, formulated as the dysconnection hypothesis of schizophrenia. Mathematical contributions include Variational Laplace, Bayesian model reduction and generalised coordinates of motion. Friston currently works on functional brain architectures and the principles that underlie self-organisation in complex systems like the brain. His main contribution to theoretical biology is a free-energy principle for open systems—and its application to action and perception, i.e., active inference.

## Introduction

The story of the development of Statistical Parametric Mapping (SPM) is—in large part—the story of modern cognitive neuroscience. The demands of the latter drove the development of the former, while reciprocally, the increased availability of scanners and analytic tools opened up new questions and fields of research.

It is remarkable how many of today’s imaging analysis methods started life in SPM, invented by Karl Friston and his colleagues, first at the Hammersmith Hospital and then at its current home in the Methods Group at the Functional Imaging Laboratory (FIL) in Queen Square. These methods include the application of the General Linear Model (GLM) and Random Field Theory (RFT) to neuroimaging, event-related fMRI, Voxel-Based Morphometry (VBM), Psychophysiological Interactions (PPI) analysis, and Dynamic Causal Modeling (DCM). These methods all rely on having a high quality of imaging registration, which has been made possible by the work of John Ashburner’s team, who have steadily developed spatial normalization (ie computational anatomy) methods with increasing sophistication: most recently Dartel, Shoot, and the MultiBrain toolbox, introduced below. Many of the same methods have found useful application to magneto/electroencephalography data (M/EEG): a particular focus for SPM development today is its application to the latest generation of MEG, which uses optically pumped magnetometers (OPMs) to record the brain in motion with exquisite sensitivity. The lead developers of SPM for MEG/EEG (M/EEG) and OP-MEG—Gareth Barnes, Vladimir Litvak, and Tim Tierney—introduce the latest developments and future directions below.

Today, there is a vibrant ecosystem of well-established neuroimaging analysis packages alongside SPM (eg AFNI, FreeSurfer, and FSL), which all have particular areas in which they specialize and excel. SPM’s focus is on technical innovations that adhere to three key principles. First, recognition that our aim as empirical scientists is to test hypotheses about the underlying causes of data, rather than just describing the data that have been observed. This mandates the use of *generative models*, which generate the data one would expect to observe, given a hypothesis, model, or mechanism; paired with suitable statistical methods for fitting and evaluating candidate models. Second, the use of carefully motivated *parametric statistics* based on rigorous mathematics that make reasonable assumptions about the data, in order to furnish fast, reproducible analyses. And third, a commitment to *open science*. SPM has been *free and open source software* since its inception, even before open science was part of our vernacular. And that philosophy has driven recent projects to make SPM more accessible, including moving development to GitHub and introducing an interface to SPM for the Python programming language (just released at the time of writing).

Although this review necessarily focusses on the core of the SPM software, we also wish to acknowledge the ground-breaking work of SPM toolbox developers in the neuroimaging community, who have built novel methods and software within the SPM ecosystem that extends its functionality. Popular examples include the SPM Anatomy Toolbox (JuBrain)—a probabilistic cytoarchitectonic atlas developed with many years of intricate anatomical work (Eickhoff et al. 2005); GIFT—a toolbox for Group ICA analysis (Calhoun et al. 2001); CONN—a toolbox for functional connectivity analysis (Whitfield-Gabrieli and Nieto-Castanon 2012); and hMRI—a toolbox for quantitative MRI (Tabelow et al. 2019). A comprehensive list of SPM toolboxes can be found at https://www.fil.ion.ucl.ac.uk/spm/ext/.

In what follows, we invited members of the SPM development team—each of whom represents a particular imaging modality or analysis method—to reflect on three questions: what were the most significant methodological and neuroscientific contributions made by SPM? Should anything have been done differently? And, what are the opportunities or plans for the future? We begin, as every SPM analysis does, with preprocessing.

### Preprocessing

Before raw scan data, such as fMRI, are analyzed, they are preprocessed. This usually starts with image registration, which involves estimating and applying a variety of spatial transformations to bring them into alignment. After this, generative models are fit to the preprocessed data to best explain how they were generated.

Because an individual’s brain remains roughly the same shape and size over the course of a study, most within-subject alignment (fMRI motion correction, and co-registration of fMRI with anatomical scans) is rigid-body. Rigidly aligning a pair of images entails finding the six parameters that maximize a measure of similarity between the images. The (negative) mean-squared difference can be used as a within-modality similarity measure for correcting head motion during a run of fMRI. This is simple, but ineffective for registering scans of different contrasts or modalities, such as aligning PET or fMRI with T1-weighted scans. An early SPM solution for inter-modal registration was a boundary-based method, whereby gray and white matter were identified in both scans, and these aligned together (Ashburner and Friston 1997).

The inter-modal registration paper included a description of a method for segmenting gray and white matter from MRI, which used Gaussian mixture models in conjunction with tissue priors. Later work involved extending this model to include deformable tissue priors and intensity nonuniformity correction—a model that we referred to as “unified segmentation” (Ashburner and Friston 2005). Before deep learning, this generative modeling became the most widely used approach in neuroimaging software, for identifying gray matter (eg for visualizing findings on cortical surfaces). For SPM users, the main use for gray matter maps was in a method known as “voxel-based morphometry” (Wright et al. 1995). This involves segmenting gray matter from a population of scans, which was then spatially normalized (see next) and blurred, before being entered into a voxel-based SPM analysis to identify anatomical differences among populations of subjects. This technique has since formed the basis of tens of thousands of papers, with variants such as voxel-based lesion-symptom mapping (Bates et al. 2003).

Image registration becomes more complicated when dealing with scans from different individuals. Not only must the differing pose of brains be handled, but also their relative shapes and sizes. In SPM, dealing with this is known as spatial normalization, and much of the evolution of SPM’s preprocessing has involved introducing more accurate ways to generate plausible brain shapes. Computers are now ~50,000 times faster than those available when SPM began in the early 1990s, in which time SPM’s spatial normalization has evolved to make use of the additional processing power and progressive increases in the spatial resolution of data. From the perspective of generative models, the model parameters required for spatial normalization are those that generate deformation fields that warp a canonical brain into a personalized, subject specific anatomy. Inverting or reversing the warp is spatial normalization.

Early SPM procedures parameterized the requisite warping fields with linear combinations of a small number of low-frequency spatial basis functions (Friston et al. 1995a). These were easy to fit using an iterative Taylor series approximation method (a.k.a., Gauss–Newton optimization), although the results often proved unstable. Slightly later, we found that better results could be obtained using more basis functions and introducing regularization into the registration model (Ashburner and Friston 1999). This was conceptualized from a maximum a posteriori perspective and was when Bayesian ideas started to creep into the software, as well as into people’s thinking about how brains might work.

The number of free parameters was still only in the order of ~1,000, partly because more flexibility would make it too easy for the one-to-one mapping (from one brain to another) to break down, precluding any inversion of the deformations. At this point, we started to consider the *inverse consistency* of the image registration problem. This work involved introducing a penalty on nonlinear deformations that should be the same when applied to the inverse of the deformations (Ashburner et al. 1999). Although this idea has since been abandoned in SPM, it was incorporated into the hippocampal subfield segmentation in FreeSurfer (Van Leemput et al. 2009), as well as the MMORF (Lange et al. 2024) spatial normalization software in FSL. SPM papers were also among the earliest to consider “groupwise” registration, whereby an average shaped template is constructed from a population of scans.

Another idea for achieving one-to-one mappings between brains was to use a scaling-and-squaring method, which involves constructing a large one-to-one deformation by repeatedly composing a smaller deformation with itself. We first mentioned this idea in the discussion of Ashburner and Friston (2005) and eventually had a working implementation in the Dartel toolbox (Ashburner 2007). While this toolbox has been widely used by neuroscientists doing VBM studies, there are now hundreds of deep learning papers about image registration, where scaling-and-squaring is used to generate deformations.

Shortly after the development of the Dartel toolbox, it was included as one of the methods in a large comparison of nonlinear image registration procedures (Klein et al. 2009). The objective was to assess which algorithms led to greater overlap of manually defined brain regions. Under the assumption that brain function is related to its structure, good performance should be assessed in terms of the overlap of BOLD “activations” over subjects; and hence greater sensitivity and specificity in a statistical analysis. Our major regret is that we asked the comparison organizers to use skull-stripped MRIs when running the SPM-based methods. Later work showed that Dartel would have comfortably beaten all the other software if the images had not been skull stripped (Ashburner and Friston 2011).

Since Dartel, there have been other incarnations of spatial normalization toolboxes in SPM, which were developed to provide more accurate measures of brain shape for studies of anatomical variability (eg Lambert et al. 2013). The first of these was the Geodesic Shooting toolbox (Ashburner and Friston 2011) and the most recent is the MultiBrain toolbox, which unifies many of the previous advances into a single model (Blaiotta et al. 2018; Brudfors et al. 2020). The latter toolbox has simplified the generation of population averaged atlases for spinal cord (Freund et al. 2022), as well as brain atlases of other species (Balan et al. 2024) or other modalities, such as CT (Sánchez-Moreno et al. 2024).

Many imaging methods will change now that deep neural networks, particularly U-Net type architectures (Ronneberger et al. 2015), have entered the scene. Given sufficient training data, these machine learning (ML) methods can be very effective, but they work in ways that are difficult to decipher. However, if we are confident that we can uncover how the human brain performs tasks, then reverse engineering ML—to recover the implicit generative model—should be trivial by comparison.

### The GLM

Following preprocessing, statistical analyses are performed. SPM brought two statistical techniques into mainstream use in neuroimaging: one known to many data practitioners, and another familiar to only a few theoretical statisticians—the General Linear Model (GLM) and Random Field Theory (RFT). Traditionally, statistical modeling tools in neuroimaging were centered around specific experimental designs, such as ANOVA or correlation. Karl Friston and Andrew Holmes recognized the value of presenting all analyses as special cases of the GLM. This approach allowed a single, general codebase to support a wide range of models. By fitting the same GLM design matrix at each voxel in a mass-univariate approach—and speeding up computation by fitting many voxels at once—SPM enabled highly computationally efficient analyses, an important consideration in the early days.

Up until SPM96, users manually created the GLM design matrix for their analysis. SPM99 introduced a model-builder that enabled users to specify the design matrix more intuitively through design specifications, albeit still requiring the user to understand their experimental design and construct linear contrasts to query their model. For fMRI data, hemodynamics were accounted for in the construction of the design matrix, by convolving user-provided stimulus timing vectors with suitable *temporal basis functions* approximating the hemodynamic response function (HRF) (Friston et al. 1994). This convolution GLM approach is now used in all major fMRI analysis packages. Importantly, methods were subsequently introduced in SPM that operate behind-the-scenes to estimate residual (unexplained) variance using a Bayesian scheme—specifically, a *variational Bayes* implementation of Restricted Maximum Likelihood (REML) (Friston et al. 2002a, 2002b). This was a landmark innovation because it enabled a single analysis framework to handle a broad class of models—from one-sample *t*-tests to repeated measures ANOVAs—while accounting for serial correlations. By decomposing the residual data covariance matrix into a weighted mixture of hypothesized matrices, the REML approach avoids ad hoc correction strategies used in other software, such as Greenhouse–Gaiser (Satterthwaite) correction*.*

Searching the brain for activations or group effects across hundreds of thousands of voxels in PET and MRI introduces a massive multiple testing problem. In the early 1990s, few robust solutions existed, and most users either ignored the problem or relied on informal methods with no guaranteed control of false positives. SPM implemented RFT results that directly addressed this challenge, providing *P*-values for voxel-wise and cluster-wise inference that accounted for image smoothness and controlled the familywise error rate. Later, methods controlling the false discovery rate were added. Notably, SPM quickly incorporated these cutting-edge inference methods into accessible software.

Since its introduction, the validity of the statistical assumptions underpinning RFT-based correction have been revisited—most recently by Eklund et al. (2016). Their results demonstrated that RFT operates correctly when SPM’s default settings are applied (see discussion and replication in Flandin and Friston (2019)). Nevertheless, some people feel more comfortable with nonparametric tests, and an SPM-compatible toolbox is available (SnPM, http://nisox.org/Software/SnPM13/). The decision not to include nonparametric methods in core SPM is a principled one, based on four arguments (Flandin and Friston 2019). First, no additional statistical sensitivity is gained by using nonparametric methods because parametric tests are statistically optimal (cf. the *Neyman–Pearson lemma*). Second, parametric tests give perfectly reproducible results, whereas the results of nonparametric tests depend on the choice of sampling strategy and the random seed used. Third, parametric tests avoid concerns about exchangeability assumptions, which complicate the use of hierarchical models that are used extensively in SPM. And finally, parametric tests run more quickly and with far less processing power than nonparametric tests. While computers are now fast enough to enable nonparametric tests in reasonable time, there is now the added concern of the climate impact of our neuroimaging analyses (Souter et al. 2025), which renews the need to minimize computational resources where possible.

Two design decisions in SPM, in retrospect, had significant consequences for practice in neuroimaging. First, the focus on the mass-univariate GLM approach was essential early on, allowing users with modest computational resources to analyze brain imaging data. However, many model extensions do not fit into a linear (convolution model) framework, limiting the flexibility and interpretability of SPM. As one example, SPM accommodates nonlinear hemodynamics via a functional Taylor expansion (a.k.a., Volterra kernels) that can be cast in terms of temporal basis functions, as mentioned above (eg Friston et al. 2000). However, this can complicate second-level analyses because region-specific responses are summarized with several parameters. A continued literature on nonlinear HRF modeling exists (Makni et al. 2005; Degras and Lindquist 2014; Chen et al. 2023), but nonlinearities in neuronal and hemodynamic responses are only dealt with explicitly in SPM by using dynamic causal modeling (DCM).

Second, SPM embraced and promoted a focus on thresholded statistical images as the primary visualization for results. While visualizing thousands of 3D input scans is impractical, a single mean percent BOLD signal image is easy to examine. The iconic SPM maximum intensity projection is an efficient representation of sparse activation results, but it can obscure issues; for instance, missing data in orbitofrontal cortex lost due to signal dropout. In contrast, software like AFNI (Cox 1996) emphasized data visualization (Taylor et al. 2023), and new inference approaches have emerged that highlight interpretable effect sizes (Bowring et al. 2019; Bowring et al. 2021). Incorporating nonlinear voxel-wise models and improved visualization are just two important directions for SPM development in future years.

Both of these aims may be gracefully accommodated under an under-used functionality within SPM known as posterior probability mapping (PPM) (Friston and Penny 2003; Rosa et al. 2010). This generalizes the mass-univariate classical inference procedures in SPM to facilitate Bayesian model comparison of any (eg nonlinear) models and presents the results in terms of the (log) evidence for different models or hypotheses. Encouraging more general uptake of the PPM approach may require providing clarification on the method’s technical foundations, particularly for users only familiar with classical (frequentist) statistics. For example, SPM requires the user to select a threshold on the *posterior log odds ratio* for visualizing results, which in turn depends on the choice of *prior* log odds ratio. While SPM provides a default value (a posterior log odds ratio of 10), more specific guidance on how to select this value for different applications would be helpful. There are also considerations regarding multiple comparisons correction. In principle, the PPM approach does not require correction because false positives do not inflate with the number of voxels, but this could be subverted by the introduction of thresholds that effectively introduce the classical notion of “significance.” We are aware of one tutorial paper on SPM’s second-level Bayesian GLM tools (Han and Park 2018) and we are currently preparing an updated primer revisiting these issues.

A potential area for future development is multivariate analysis. Recently, there has been increased interest in testing for experimental effects that are expressed in multivariate spatial patterns over the brain (sometimes called *representations*). The standard generalization of the GLM to multivariate data is called Canonical Variates Analysis (CVA). A software tool for CVA was implemented early in the development of SPM (Friston et al. 1995b), which remains available via a button in the graphical interface; however, this feature is not widely used. There may be an opportunity to increase the uptake of this approach, simply by improving the tool’s graphical output and documentation. More recent extensions of CVA could also be incorporated, which have refinements such as sparsity constraints that provide more readily interpretable results—see Zhuang et al. (2020) for a comprehensive review. The need for improved, well-grounded multivariate methods is particularly timely due to recent widespread adoption of multivariate analysis techniques that either preclude fully modeling factorial experimental designs (distinguishing main effects, interactions, and noise) or that use test statistics such as *classification accuracy*, which are provably less sensitive (or at most, equally sensitive) as standard statistical tests, as per the *Neyman–Pearson lemma*. Relevant developments in SPM’s multivariate analyses to date comprise Multivariate Bayes, a decoding model for fMRI data (Friston et al. 2008a), and more recently *variational Representational Similarity Analysis*, which was first proposed by Friston et al. (2019) and is currently being adapted for application to M/EEG data.

In short, SPM’s introduction of GLM-based modeling and topological (RFT-based) inference fundamentally shaped the field of neuroimaging, providing a standard model that remains largely unchanged since its inception. Developing the next generation of multivariate analysis techniques is a current priority. While the GLM was originally introduced for PET and MRI data, it was later expanded to EEG/MEG, which we turn to next.

### SPM for M/EEG

EEG/MEG functionality was introduced in SPM5 through a series of papers exploring the application of the SPM framework to event-related potentials (Kiebel and Friston 2004a, 2004b). When SPM for M/EEG was first presented to an external audience at the May 2005 SPM course, its three main components had already been established: (1) the extension of the topological inference framework, based on the General Linear Model and Random Field Theory–based correction for multiple comparisons, to scalp × time and time × frequency data (Kilner and Friston 2010); (2) an empirical Bayesian source reconstruction framework using canonical anatomy (Friston et al. 2006; Mattout et al. 2007); and (3) Dynamic Causal Modeling for Evoked Responses, pioneered by Olivier David (David and Friston 2003; David et al. 2005; David et al. 2006; Kiebel et al. 2006).

The SPM8 release introduced several improvements. A collaboration with the developers of the FieldTrip toolbox (Oostenveld et al. 2011), based at the Donders Institute in Nijmegen, was established, integrating FieldTrip code into SPM to support key functionality, including compatibility with multiple raw data formats, digital filtering, spectral analysis, and electromagnetic forward modeling. A new data format leveraging MATLAB’s object-oriented features was introduced, facilitating automatic integrity checks and greatly enhancing code stability. The transition to SPM12 involved a further overhaul of M/EEG functions, providing a unified batch interface for pipeline building.

We now review the methodological contributions of SPM to M/EEG analysis in greater detail. A key advancement for topological inference was conceptualizing M/EEG data as a particular case of continuous 2D or 3D images. This is logical, as M/EEG sensors provide spatial sampling of continuous electric potential and magnetic field patterns. By converting sensor data into 2D (time × frequency) or 3D (scalp × time, scalp × frequency) arrays, SPM enabled statistical comparisons across conditions and groups using the familiar GLM framework. A notable strength of this framework is its ability to accommodate complex experimental designs, including multiple regression, factorial designs, and hierarchical structures with both within- and between-subject factors. This approach supports traditional factorial studies common in clinical and psychological research (Pegg et al. 2020; Yao et al. 2021), as well as model-based studies examining correlations between hidden model variables and evoked activity; including the effects of pharmacological interventions (Weber et al. 2020; Hein et al. 2021). Another novel GLM-based application in SPM is convolution modeling (Litvak et al. 2013; Jha et al. 2015), which allows for the separation of temporally overlapping responses and the estimation of temporo-spectral impulse response functions for continuous stimuli. This method has since been applied to evoked activity (Spitzer et al. 2016) and further developed outside SPM in the “Unfold” toolbox (Ehinger and Dimigen 2019; https://www.unfoldtoolbox.org/).

Despite these strengths, the topological inference approach in SPM has certain limitations. Historically, SPM was designed for analyzing 3D brain images, restricting its support to data with up to three dimensions. This is despite the fact that Random Field Theory theoretically applies to any dimensionality and M/EEG research sometimes entails higher-dimensional data, such as whole-brain time–frequency data in source space. However, such analyses are uncommon even in toolboxes that support them (eg FieldTrip) due to challenges in visualization and interpretation of the results, as well as the highly conservative multiple-comparison corrections required. Another issue, highlighted by Eklund et al. (2016), is the potential inflation of false-positive rates in cluster-level inference unless an appropriate conservative cluster-forming threshold is used (typically *P* < 0.001, the default in SPM). This can be inefficient for sensor-level and time–frequency M/EEG data, where large clusters of voxels with relatively weak effects are often of interest. However, these constraints could potentially be relaxed under specific conditions common in M/EEG analysis, which we are currently working to characterize.

A significant contribution from the SPM team and collaborators was the first open multimodal, multisubject dataset for a cognitive study, acquired and published by Wakeman and Henson (2015), coinciding with the emergence of the open science movement. The full analysis pipeline for this dataset, originally included in the SPM12 manual and later published by Henson et al. (2019), inspired similar efforts from other academic software developers. This culminated in the Frontiers research topic “From Raw MEG/EEG to Publication: How to Perform MEG/EEG Group Analysis with Free Academic Software” (Delorme et al. 2022), which featured 25 papers based on open data and code, 10 of which analyzed the Wakeman and Henson dataset.

The M/EEG inverse problem is ill-posed, meaning that different mixtures of neural sources can give rise to similar data. Solving this problem therefore depends on additional assumptions to explain the current flow in the brain given the measured fields. One of the main contributions of SPM to the M/EEG literature is the use of generative models (models which can generate data) and the introduction of a cost-function to quantify the relative probabilities of competing models (or prior sets of assumptions). This cost-function makes use of negative variational free energy as a proxy for model evidence (Hinton and Van Camp 1993).

Another unique aspect of SPM’s source analysis approach is the use of canonical anatomy (Mattout et al. 2007). This builds on SPM’s unified segmentation and normalization framework (Ashburner and Friston 2005) to inverse-transform a single set of head and cortical meshes to fit individual anatomies. This method is highly robust and performs well even with low-quality MRI scans. Moreover, it elegantly addresses the challenge of group analysis, as results on the cortical mesh can be mapped to template anatomy, due to the one-to-one correspondence between individual and template mesh vertices.

A common prior assumption used to solve the M/EEG inverse problem is that the data are generated by a small (typically < 10) number of current sources or dipoles. Kiebel et al. (2008b) expressed the dipole fitting problem within a Bayesian framework. This enabled the user to make quantitative statements against any fit parameter; eg how many dipoles might explain the data. These dipoles form the spatial part of the spatiotemporal models underlying Dynamic Causal Modeling (DCM) for M/EEG (Kiebel et al. 2009).

Another common approach to solve the inverse problem in the M/EEG literature is the use of distributed source models. These models of multiple (typically > 100) sources are used to describe the distribution of the current flow generating the M/EEG data across the cortical manifold (Hämäläinen and Ilmoniemi 1994; Pascual-Marqui et al. 1994). Each of these inversion methods has its own implicit assumptions, which are embodied in the structure of the source covariance matrix (Mosher et al. 2003). Friston et al. (2008b) introduced an empirical Bayesian scheme to quantitatively compare between traditional M/EEG prior assumptions and added a new approach known as Multiple Sparse Priors (MSP). MSP makes use of patches (or collections of simultaneously active patches) of cortex which can be used to create specific sensor-level covariance components. These modeled sensor-level covariance components are then weighted, mixed, and refined to give an estimate of the empirically observed sensor-level covariance (using model evidence as a cost-function). A later addition to the suite of prior assumptions was the Empirical Bayesian Beamformer (Belardinelli et al. 2012). This made use of beamformer assumptions of Van Veen et al. (1997) to generate a single source covariance prior.

The utility of the model evidence framework was demonstrated by Henson et al. (2009)—comparing forward models—making use of generic or individual anatomy. This was followed by work showing how other modalities (like fMRI) could provide empirical priors for MSP (Henson et al. 2010). Importantly, these priors could be discarded if they did not increase the marginal likelihood of the solution.

Anatomy was revisited by López et al. (2012), where the problem of estimating the location of the brain—given only the MEG data—was addressed. This is a challenging estimation problem as the lead-fields (impact of current flow on the sensors) depend nonlinearly on the orientation and location of the brain (and surrounding tissue). Model evidence was maximized when the brain was located in the correct position. This covariation between precise anatomy and higher model evidence scores was later exploited in Stevenson et al. (2014) and Little et al. (2018) in which progressively more distorted anatomical models gave rise to lower model evidence. The anatomy acts as a ground truth and the decrease in model evidence (negative variational free energy), as the cortex distorts, anchors the formal model comparison metric (without knowledge of the true current distribution) to reality. Another perspective on the same idea was to estimate the true shape of the subject’s cortex that gave rise to the measured MEG data (López et al. 2017).

These ideas led to a series of papers probing the spatial resolution available from MEG data. In these studies, authors would typically compare cortical manifolds defined by either the pial or white matter surface (ie lower or upper layers of the cortex). Initial simulation studies (Troebinger et al. 2014; Bonaiuto et al. 2018b) led to empirical work using head-casts to demonstrate that (in accordance with computational models and invasive recordings) current flow fluctuations within different frequency bands could be shown to derive from distinct layers of cortex (Bonaiuto et al. 2018a).

More recently, systems based around optically pumped magnetometers (OPMs)—which do not require cryogenic cooling—have become available (Tierney et al. 2019). The same SPM machinery can be used to process these data, and there are multiple exciting technical challenges which present themselves. These range from co-registration (Duque-Muñoz et al. 2019) to optimal array design (Tierney et al. 2020); and to explore how useful presurgical OPM estimates in epilepsy might augment anatomical lesion data (Mellor et al. 2024).

### Optically pumped magnetometers

Magnetoencephalography using OPMs, which do not require cryogenic cooling, are an increasing focus of the SPM team (Tierney et al. 2019). These sensors provide increased signal and spatial resolution when compared to their cryogenic counterparts (Boto et al. 2016; Iivanainen et al. 2017; Iivanainen et al. 2021; Wens 2023). They are also sufficiently lightweight that fully wearable MEG systems are now a reality (Schofield et al. 2024). This is of particular relevance to applications such as surgical planning in epilepsy (Vivekananda et al. 2020; Hillebrand et al. 2023), essential tremor (West et al. 2025), and neurophysiological investigations of young children (Feys et al. 2022; Rhodes et al. 2024; Vandewouw et al. 2024; Corvilain et al. 2025). In all these cases, motion of the participant can be a significant confound. Having a fully wearable brain imaging system can help mitigate this issue as the sensors themselves are fixed with respect to the brain. There is therefore minimal signal distortion due to movement (Boto et al. 2018; Seymour et al. 2021; O’Neill et al. 2025). A further advantage of OPM systems is that their placement can be made application specific. This flexibility in design means that systems can be optimized to target challenging brain structures such as the cerebellum or hippocampus (Lin et al. 2019; Tierney et al. 2021), the spinal cord (Mardell et al. 2024), or even the muscles of individual digits (Kruse et al. 2025).

While these emerging applications are very exciting, there are a number of unique challenges that are associated with writing code for use in OPM research. Essentially, there are qualitative differences between arrays of OPMs and arrays of cryogenic MEG sensors beyond their wearability. For instance, the vast majority of cryogenic systems rely on gradiometers rather than magnetometers (Vrba and Robinson 2002) because of their excellent ability to elude environmental interference. While it is possible to develop optically pumped gradiometers (Limes et al. 2020; Nardelli et al. 2020; Cook et al. 2024), they often compromise on white noise, wearability, or vector measurement. This is by no means the only difference and the sheer variety of OPM systems available (Colombo et al. 2016; Limes et al. 2020; Alem et al. 2023; Gutteling et al. 2023; Cook et al. 2024; Schofield et al. 2024) make preparing software suitable for all systems and applications a substantial challenge. For instance, the number of channels, vector measurements, dynamic range, closed loop implementations, bandwidth, and data acquisition systems can vary dramatically from laboratory to laboratory. This was a strong motivation to develop OPM simulation tools that would allow us to explore the impacts of such system design features (Tierney et al. 2020). These tools have been used in applications such as exploring the possibility of laminar level resolution recordings with OPMs (Helbling 2025), selecting necessary channel numbers and types for interference mitigation (Tierney et al. 2022) and also fusion of structural and functional data in clinical contexts (Mellor et al. 2024).

As previously noted, magnetometers have limited capacity to suppress environmental interference and gradiometers are often preferred (Hämäläinen et al. 1993; Vrba and Robinson 2002). However, there are many software frameworks that could be used to attain gradiometer-like performance from arrays of magnetometers. These include data-driven approaches such as SSP (Uusitalo and Ilmoniemi 1997), ICA (Vigârio et al. 2000), and beamformers (Brookes et al. 2021). While such approaches are of great utility, their use can be difficult to generalize when the sensors, their number, environment, and degree of magnetic shielding vary so wildly from study to study (Iivanainen et al. 2019; Zhang et al. 2020; Holmes et al. 2022; Seymour et al. 2022; Bardouille et al. 2024; Holmes et al. 2024). Such concerns were the driving force behind adopting a model-based approach to interference mitigation in SPM where performance could be generalized across systems while being minimally dependent on the data.

The first algorithm proposed was termed homogeneous field correction (HFC) and was designed for use on systems with very few channels and vector measurements (Tierney 2021). It produces a spatial response function similar to a gradiometer with a long baseline (Vrba and Robinson 2002) and importantly it does not suffer the same white noise increase observed in synthetic gradiometers (Fife et al. 1999; Nardelli et al. 2020). Furthermore, the algorithm’s performance is stable with as few as 10 channels (Tierney et al. 2022) and resultingly has seen application across many contexts such as surgical planning in epilepsy (Hillebrand et al. 2023), harmonization of multisite studies (Hill et al. 2022), and in comparisons with cryogenic MEG systems (Rhodes et al. 2023).

The simplicity of HFC and its ability to be used with highly variable systems with very few sensors is certainly a key strength. However, it does not take advantage of the information provided by large channel systems (Pratt et al. 2021; Alem et al. 2023; Seedat et al. 2024) to model spatially complex interference, reduce white noise by oversampling, and provide robust modeling in the presence of sensor nonlinearities (Taulu and Simola 2006; Borna et al. 2022). Therefore, we considered how existing model-based approaches such as SSS (Taulu et al. 2005; Taulu and Kajola 2005) could be harnessed in SPM (Oswal et al. 2024). Unfortunately, the method requires that the OPM system has been optimized to enhance the stability of the method (Nurminen et al. 2023; Wang et al. 2023; Zhdanov et al. 2023) or that the user introduces a data-driven component to the method (Holmes et al. 2023). We sought to eschew these issues by introducing an adaptive multipole model based on spheroidal harmonics and orthogonal projections (Tierney et al. 2024) which would have guaranteed stability across any OPM system without requiring that the system itself be optimized. One might argue such an approach could be widely applicable across the wide variety of OPM systems that are becoming available to the Neuroimaging community.

While great progress has been made on interference mitigation, there are outstanding challenges. Most notably the issue concerning how we analyze sensor-level data in the most statistically powerful way possible. While source modeling can take advantage of existing SPM machinery, it is not so straightforward at the sensor level. Our statistical inference schemes capitalize on the spatial smoothness of data to maximize statistical power (Worsley et al. 1996; Barnes et al. 2011; Barnes et al. 2013). This constraint also applies to various forms of nonparametric inference (Mensen and Khatami 2013). However, many OPM systems will produce field patterns that are not smooth or continuous, limiting statistical power at the sensor level. This is because spatially proximal OPM sensors may measure the magnetic field in different, or even orthogonal, directions. These configurations are often motivated by practical considerations such as wire paths or theoretical reasons such as optimal spatial sampling and interference mitigation (Schoffelen et al. 2025). Considering this tension between statistical power, system design, and optimal spatial sampling, new modeling approaches may be required to create statistically interpretable OPM fields (Cai et al. 2025) and standardize their layouts (Alexander Nicholas et al. 2025).

### Connectivity

Standard mass-univariate SPM analysis of functional neuroimaging data, of the sort described above in the context of fMRI and M/EEG data, is invaluable for localizing experimental effects in the brain, but it makes a strong and unrealistic assumption. It treats the voxels or channels as separate units, driven only by the neurons located within the local patch of brain tissue. In reality, neural activity depends on afferent connections from other brain regions, which calls for connectivity to be included in models of neural responses.

The guiding principle behind connectivity analysis in SPM is to recognize that *effective connectivity*—the time-dependent, directed influences among neural populations —mediates the underlying causes of our functional neuroimaging data (as well as downstream derivatives of that data, such as *functional connectivity*). We therefore face a modeling problem: how to infer effective connectivity, which we cannot directly observe, from noninvasive functional measures like fMRI or M/EEG.

The first step toward estimating effective connectivity in SPM was the introduction of Psychophysiological Interaction (PPI) analysis using fMRI data (Friston et al. 1997). Here, each voxel’s timeseries is explained in terms of a two-factor design: the main effect of a cognitive task, the main effect of a seed region’s BOLD fMRI timeseries, and their interaction, which is referred to as the PPI. The original PPI paper has been cited thousands of times, reflecting its widespread uptake in cognitive neuroscience and its re-implementation in multiple software packages, including popular toolboxes for SPM called Conn (Whitfield-Gabrieli and Nieto-Castanon 2012) and gPPI (McLaren et al. 2012).

While PPI analysis is simple and has the advantage that it generates statistical maps spanning the entire brain, its major limitation is that it cannot capture reciprocally connected brain networks with multiple regions. This motivated the development of Dynamic Causal Modeling (DCM), which was initially introduced for fMRI data (Friston et al. 2003) and subsequently expanded to include biologically plausible *neural mass models* of MEG/EEG/LFP data (Moran et al. 2013). More recently, DCM has been finding applications beyond neuroimaging (eg epidemiology; Friston et al. 2020). DCM builds upon two well-established sets of technical methods. The first are *state-space models*, which come from the engineering field of control theory. State-space models are used to formally describe neural dynamics and how they generate neuroimaging data (making DCM a *generative modeling* approach). Crucially, DCM pairs these state-space models with the necessary statistical tools to assess how well they explain the data, using an approach called *variational Bayesian inference*. Variational Bayes was originally introduced in statistical physics and later developed in machine learning, and is used in DCM to assign a statistical score to each candidate model, called the log-evidence.^1^ DCM employs a generic variational Bayes scheme called Variational Laplace (Friston et al. 2007; Daunizeau 2017; Zeidman et al. 2023), which assesses the approximate log-evidence for a broad class of models, giving reproducible results without requiring time-consuming numerical sampling methods. Hypotheses are tested by comparing the approximate log-evidence for different candidate models, a procedure called *Bayesian model comparison*.

A key contribution of DCM has to been to democratize access to mathematical models of neural connectivity. A range of well-established models are provided with the SPM software and can be configured using a graphical user interface, without mathematical expertise. In tandem, the Variational Laplace scheme for fitting models is agnostic to the specific model being used, enabling new models to be implemented easily. This has driven the development of models with increasing sophistication. For example, the Canonical MicroCircuit model (Bastos et al. 2012) was a particularly important milestone because it was the first model to distinguish the parallel ascending and descending streams of neural activity that are required for *predictive coding* accounts of brain function. *Neural field models*, which capture the spatial extent of neural processes across the cortical sheet, are another particularly advanced application of DCM (Pinotsis et al. 2012).

In parallel with the development of neural models, there has been continuous development of the technology for model inversion and statistical testing in DCM. Soon after its introduction, DCM was expanded for fitting spectral data rather than timeseries, enabling applications to M/EEG data in the frequency domain (Moran et al. 2007) and, more recently, to resting-state fMRI data (Friston et al. 2014).

Methods for between-subjects analysis have also evolved. Thousands of studies were enabled by a framework called Random Effects Bayesian Model Selection (RFX BMS), which estimates the proportion of people in a population whose data would best be explained by each candidate model (Stephan et al. 2009). A more recent development has been the Parametric Empirical Bayes (PEB) framework, which treats the individual model parameters—for example, individual neural connections—as *random effects* that are sampled from the population (Friston et al. 2016). This provides a straightforward way of testing whether different mixtures of parameters can explain commonalities and differences between people, in terms of either discrete group differences or parametric effects, such as clinical scores. Finally, a particularly important statistical innovation, which underwrites the PEB approach, is *Bayesian Model Reduction* (formally referred to as *post-hoc DCM*) (Rosa et al. 2012; Friston et al. 2016). This is an analytic procedure for approximating the log-evidence and parameters of *reduced* models—which have certain mixtures of parameters turned off—given a full model. This technology enables the evidence for large numbers of models to be scored in a matter of milliseconds or seconds on a modern computer, without risking each model-fitting falling into distinct local optima.

In the future, we can expect new applications of DCM for clinical research, as it enables explainable, biologically meaningful parameters to be identified from neuroimaging data. In the case of fMRI, this is likely to be supported by improved hemodynamic models that have been proposed for standard-resolution and laminar fMRI (Uludağ 2023). To give a compelling recent example of a clinical application of DCM, Ereira et al. (2024) evaluated the use of DCM with resting-state fMRI for early detection of Alzheimer’s disease. They fitted DCM connectivity models to resting-state fMRI data from 81 individuals who were subsequently diagnosed with Alzheimer’s disease in the nine years following imaging, as well as 1,030 matched controls. They applied a machine learning model (elastic-net logistic regression) to predict future incidence and time to diagnosis using the estimated DCM connectivity parameters. They were able to predict both future incidence and time to diagnosis, with greater accuracy than structural connectivity, functional connectivity, or behavioral measures. This study demonstrated the predictive validity of DCM connectivity parameters in the context of disease, and the principle that model parameters—such as those from a DCM—tend to more readily support a separation of groups of people into diagnostic groups than the raw data itself (Brodersen et al. 2011).

DCM sits at the intersection of neurobiology, engineering mathematics and (variational) Bayesian statistics. Leveraging all of these fields is both its key strength and, perhaps, its greatest challenge. There is a common perception that DCM is complicated and inaccessible, which is particularly problematic if people cannot properly report their results or understand the modeling assumptions therein. To address this, significant work has been invested in recent years to write tutorial papers explaining the theory and application of DCM (eg Henson et al. 2019; Zeidman et al. 2019a, 2019b; Novelli et al. 2024). Nevertheless, there is still room for improvement with regard to documentation and training, and this is a priority for the SPM team going forwards, for instance through the ongoing development of a new documentation website (https://www.fil.ion.ucl.ac.uk/spm/docs/tutorials/).

### Behavioral modeling

So far, we have focused upon the idea that, as neuroscientists, we measure brain activity and use generative models to solve inverse problems to investigate the anatomy and physiology that cause those data. Interestingly, this closely mirrors the problem that the brain itself must solve and suggests we can apply the same tools—developed for the analysis of imaging data—as models of how our brains interpret the data they acquire via the eyes, ears, and other sensory organs (Friston et al. 2012).

Active Inference has developed into a popular theoretical framework (Parr et al. 2022), largely supported by extensive software demonstrations in SPM’s DEM toolbox. Early software implementations were based upon some of the same (generalized) filtering schemes developed for stochastic DCM (Friston et al. 2010)—which themselves resemble the distributed error-minimization associated with predictive coding theories of brain function (Srinivasan et al. 1982; Rao and Ballard 1999; Friston and Kiebel 2009). The core idea is that when one allows for action that can change the data-generating process, one can maximize Bayesian model evidence both by fitting the model to data and by fitting the data to the model (Hohwy 2016). While this sounds a little abstract, in practice this means equipping model inversion schemes with something akin to a spinal or brainstem reflex arc whose setpoint is the proprioceptive afferent signal predicted by the model (Adams et al. 2013a). By correcting deviations from this setpoint via simple negative feedback loops, one can generate continuous behavioral trajectories. Applications of the above range from models for complex motor control (Friston et al. 2011; Parr et al. 2021), psychosis (Adams et al. 2013b), communication in songbirds (Friston and Frith 2015), to cerebellar eyeblink conditioning (Friston and Herreros 2016). Each of these problems is formulated in terms of the underlying generative models that the brain solves, which means that apparently disparate behaviors can be characterized using the same formal language.

A further development came from bringing experimental design principles more directly into this form of modeling. Appealing to theories of optimal experimental design (Lindley 1956; MacKay 1992), and combining these with ideas from expected utility theory (Wald 1947; Todorov 2009), one can prospectively evaluate the prior plausibility for different courses of action that bring about informative sensory data that are consistent with preferred (or “rewarding”) outcomes. This led to the development of a suite of tools for simulating behavior through the variational inversion of Partially Observable Markov Decision Processes (POMDPs) (Da Costa et al. 2020). POMDP generative models for Active Inference are typically formulated in terms of categorial variables, which support the process of decision-making and planning; affording the opportunity for the study of curious behavior (Friston et al. 2017b; Schwartenbeck et al. 2019)—a key theoretical emphasis that distinguishes this approach from reinforcement-learning approaches that prioritize (reinforceable) reward-driven behavior (Sutton and Barto 1998). In effect, the use of categorical variables reformulates the problem of planning as one of model selection (Parr and Friston 2019), where we must compare alternative models of the future conditioned upon different courses of action.

The continuous filtering and the categorical POMDP formulations each provide simple units from which more sophisticated models can be built. One of the most obvious examples of this has been the development of hierarchical models—characterized by a factorization of timescales (Kiebel et al. 2008a; Friston et al. 2017c). Deep temporal models use filtering or POMDP models with slow dynamics to provide empirical priors for models with faster dynamics. For instance, one could use the slower dynamics associated with the semantic and syntactic structures of a sequence of sentences to provide priors for the (faster) sequences of words in each of those sentences. One can go further and “mix-and-match” the two different generative model architectures such that a higher-level POMDP model facilitates decision-making that can be implemented via the continuous dynamics of a lower-level filtering model (Friston et al. 2017a; Parr and Friston 2018b). In principle, these models can be combined with arbitrary generative models, as has recently been demonstrated in the context of bespoke speech-recognition schemes (Friston et al. 2021b).

In addition to theoretical development, behavioral modeling schemes of this sort have been used for empirical neuroscience. Some of these approaches involve using theoretical models to formulate empirical predictions. For instance, a theoretical model developed to understand saccadic sampling patterns in visual neglect (Parr and Friston 2017) was used to motivate hypotheses about effective connectivity in healthy controls, subsequently assessed using DCM for MEG (Parr et al. 2019b). Another example is the use of a model of the belief-updating that supports decision-making to develop parametric regressors to assess hypotheses about functional anatomy using fMRI (Schwartenbeck et al. 2015). However, one can go further than this and use the variational inversion schemes available in SPM (Zeidman et al. 2023) to fit behavioral models—including, but not limited to, Active Inference models—directly to behavioral data (Schwartenbeck and Friston 2016). This can be thought of as a form of behavioral DCM, in which choice data or movement trajectories take the place of functional imaging timeseries. A couple of examples of this include the modeling of smooth pursuit eye movements with occlusions to assess the precision with which people predict the movement of a visual target (Adams et al. 2015), and the modeling of discrete choices to assess the degree to which behavior is driven by the imperative to resolve uncertainty (Mirza et al. 2018). Such approaches, where the experimenter’s generative model includes the inversion of the subject’s generative model, are sometimes referred to as meta-Bayesian inference (Daunizeau et al. 2010); ie observing the observer.

With the growth of this field, there are many different future directions developing in both neurobiology and artificial intelligence. Some of the important developments to look out for include (1) the scaling of current approaches to deal with naturalistic settings beyond those of minimal proofs-of-principle and (2) a closer integration between models of behavior and more physiological (DCM-like) generative models. Emerging developments in the scaling domain include structure learning (Smith et al. 2020; Friston et al. 2024), where generative models may be built up over time as supported by the sensory data one observes. This can be approached both through “pruning” of overparameterized models through Bayesian model reduction (Friston et al. 2018) or through adding to models when additions favor an increase in accuracy relative to any increased model complexity. Further scalability involves optimization of the process of planning, to deal with the complexity emerging from long sequences of decisions. Recent developments using inductive (Friston et al. 2025) and sophisticated (ie recursive) (Friston et al. 2021a) schemes have greatly improved the performance and scope of Active Inference models.

Finally, the integration between behavior and physiology offers another exciting avenue. Recent theoretical work has considered the anatomy needed by the brain in order to invert its generative model and perform belief-updating (Parr and Friston 2018a; Parr et al. 2019a) and physiology (Friston et al. 2017a). This raises the possibility that one might develop generative models predictive of both behavioral and functional imaging timeseries, enabling a form of Bayesian fusion in which both data modalities inform inferences about the same underlying model.

### Open science

SPM’s continuous development over the past three decades represents more than analytical progress in neuroimaging: it reflects a commitment to open scholarship that has helped shape the field. Before the terms *open science* and *reproducibility crisis* became prevalent in the scientific lexicon (Ioannidis 2005; Open Science Collaboration 2015; Gorgolewski and Poldrack 2016), SPM adhered to principles that would later become foundational of the broader open scholarship movement.

Since its inception, SPM has been made freely available to the neuroimaging community (Ashburner 2012). By providing open distribution and full access to its codebase, SPM was designed to foster collaboration, facilitate rigorous scrutiny of methods, and establish a standardized yet adaptable analysis framework across laboratories. SPM’s open-source philosophy continues to drive its evolution, most notably with the recent transition of its development to GitHub (Tierney et al. 2025). This move enhances transparency, democratizes neuroimaging methods developments, and encourages broader community involvement (https://github.com/spm; https://www.fil.ion.ucl.ac.uk/spm/docs/development/).

True openness in software extends far beyond simply making code available: it requires comprehensive, transparent documentation of the underlying algorithms, statistical models, and their implementation. This ensures that users not only have access to the tools but also possess the knowledge to apply them correctly and adapt them as needed. To this end, SPM provides extensive user manuals (https://www.fil.ion.ucl.ac.uk/spm/docs/reference/), online tutorials (https://www.fil.ion.ucl.ac.uk/spm/docs/tutorials/), methodological papers, and books (eg Penny et al. 2011). Beyond formal documentation, SPM fosters a culture of openness by providing multiple avenues for ad hoc support and knowledge exchange. The SPM mailing list (https://www.fil.ion.ucl.ac.uk/spm/support/) is actively monitored, offering a space where users can ask questions, discuss methodological challenges, and seek guidance from both developers and the wider community. Complementing this, weekly meetings of the FIL Methods Group serve as an interactive forum where researchers from any institution can ask to present their projects and receive feedback on SPM-related issues. These initiatives ensure that users at all levels have access to expert advice, fostering an environment of collaboration and shared learning.

SPM also places a strong emphasis on education through its regular international courses, which provide comprehensive training in both foundational principles and advanced neuroimaging methods. Covering a range of modalities, including (f)MRI, EEG, MEG, and OP-MEG, these courses equip researchers with both theoretical knowledge and practical statistical analysis skills. Crucially, all teaching materials are openly available and supplemented with interactive web-based tutorials, enabling self-paced learning and facilitating broader dissemination of best practices in neuroimaging analysis (https://www.fil.ion.ucl.ac.uk/spm/docs/courses/).

Reflections on SPM’s contributions to neuroimaging and the wider open scholarship movement also bring to light gaps that have emerged over decades of development. A commonly raised concern is SPM’s reliance on MATLAB, a commercial programming environment, which due to licensing costs and paywalls can restrict access. The choice of MATLAB, however, was well suited to the early 1990s, when SPM was first starting, providing one of the most powerful and accessible platforms for numerical computation at the time. However, the growing availability and computational power of open-source languages, such as Python, has led to increasing calls for a MATLAB-independent version of SPM. While a compiled standalone version of SPM is already available and does not require a MATLAB license to run (optionally in ready-to-use Docker and Singularity containers), full integration with an open-source language represents the next logical step. Tools like Nipype currently allow Python users to incorporate SPM into their workflows (Gorgolewski et al. 2011), but the long-term goal is to deliver a seamless, native SPM experience in both MATLAB and Python environments. Development of SPM’s Python interface, spm-python, is well underway (https://github.com/spm/spm-python).

Looking ahead, several opportunities for fostering open science and reproducibility could shape SPM’s development. One major priority is deeper integration with the Brain Imaging Data Structure (BIDS) standard (Gorgolewski et al. 2016). BIDS provides a consistent framework for organizing and describing neuroimaging data, facilitating data sharing, reusability, and transparency in analytic workflows. Strengthening compatibility with BIDS would streamline the incorporation of SPM into open data repositories such as OpenNeuro (https://openneuro.org), enabling seamless sharing of both raw data and analysis pipelines. This alignment would represent a significant step toward achieving true computational reproducibility.

In parallel, containerization technologies offer promising avenues for encapsulating SPM analyses within complete, portable computational environments. Packaging analyses in containers ensures they remain reproducible over time, regardless of changes in software dependencies or operating systems. Moreover, containerized pipelines could serve as reference implementations, reducing variability introduced by differing local configurations and promoting more standardized preprocessing and statistical workflows (Nichols et al. 2017; Renton et al. 2024).

We also recognize the importance of effective community engagement as the field evolves. While the SPM mailing list has long served as a valuable forum for support and discussion, future improvements should include more dynamic and responsive platforms for communication, such as dedicated community forums or collaborative issue-tracking systems, to streamline feedback, address user queries more efficiently, and foster a more interactive development environment.

The future of SPM’s development will build on its legacy of open scholarship. By continuing to prioritize transparency and methodological rigor, SPM can help neuroimaging research navigate the challenges of reproducibility and accelerate scientific discovery through open knowledge sharing. These principles remain as relevant today as they were three decades ago—a testament to how forward-thinking the software’s original design was and a framework for its continued development in the decades to come.

### Coda

In short, the story of SPM mirrors the story of cognitive neuroscience. One could argue that imaging neuroscience was the catalyst that converted cognitive science into cognitive neuroscience; simply because neuroimaging brought neuroanatomy, neurophysiology, and neuropsychology to the table when addressing questions about cognitive or functional anatomy; eg structure–function relationships and all that entails. SPM mirrors the conceptual developments over the past 30 years of cognitive neuroscience, trying to operationalize, formalize, and democratize the remarkable advances we have witnessed over that time.

SPM heralded the era of open science in which we currently find ourselves. The first release of SPM coincided with the launch of the Human Genome Project (Lander et al. 2001) and the inception of large-scale data sharing that foreshadowed the era of big data. Notably, the Human Genome Project inspired initiatives like BrainMap in imaging neuroscience (Laird et al. 2005). It is interesting to reflect that at the time of SPM’s release, brain imaging data were much larger than anything that had been seen in the life sciences previously. In some respects, this explains the early focus on solving the multiple-comparison problem and the emergence of random field theory that underpins topological inference—an approach adopted by the astrophysics community (Bond and Efstathiou 1987).

However, SPM—as a poster child for open science—was not simply a reflection of its availability. The key move was to engage the brain imaging community through SPM toolboxes, interoperability—eg with Brainstorm (Tadel et al. 2011)—and an operational basis for establishing common standards and common ground. Establishing common ground was particularly important in the early days of brain mapping because neuroimaging had to establish its integrity and validity as a new and unproven discipline. This put a certain pressure on the rigor that underwrites the procedures offered by SPM. Without exception, these procedures are predicated on a simple imperative: the analysis procedures are there to enable people to ask questions of their data. Formally, this is articulated in terms of classical or Bayesian model comparison, which means there must always be a generative model under the data. A commitment to generative modeling has endured for three decades: from early linear convolution models for fMRI through to expressive dynamic causal models for MEG. There are two interesting aspects of this commitment.

The first reflects an inclusive aspect of SPM development; namely, its interdisciplinary aspect. A nice example of this was the co-location of the FIL—where much of the foundational software was developed—and the Gatsby Computational Neuroscience Unit in Queen Square, directed by Geoffrey Hinton. Machine learning and computational neuroscience was, at that time, committed to generative models as the basis of enabling technology and accompanying software (Hinton and Zemel 1993; Dayan et al. 1995; Neal and Hinton 1998; Hinton 2022). One could argue that work of Hinton and colleagues led to the kind of *mortal computation* seen in the brain (Hinton 2022) and, ultimately, to generative AI we enjoy, some 30 years later. Having said this, a commitment to generative modeling means that SPM is something of an exclusive club, in that it precludes technology that does not support explanations (eg interpretable AI) or hypothesis testing (eg model comparison). In this sense, it is unlikely that SPM will embrace or endorse any machine learning or related approaches that are quintessentially descriptive, correlative, or classification based.

Another example of cross-disciplinary inspiration speaks to the 30th birthday of *Cerebral Cortex*: the first paper of the first issue of *Cerebral Cortex* was a seminal paper by Felleman and Van Essen (1991), which established hierarchical brain architectures as a fundament of functional integration in the brain. This paper was (literally) iconic and has remained so through the ensuing eras of connectomics and network neuroscience: eg Bullmore and Sporns (2009); Bassett and Sporns (2017). So, why is this relevant for SPM?

The co-construction and development of SPM reflects the focus on two key principles of functional anatomy; namely, *functional segregation* and *functional integration*. Early characterizations of brain mapping—in terms of neo-phrenology—speak to the importance of mapping functionally specialized yet anatomically segregated brain regions as a prelude to asking deeper questions about the distributed processing and implicit connectivity: eg Zeki and Shipp (1988); Mesulam (1998); Hilgetag et al. (2000); Amunts et al. (2019). The shift in focus from mass-univariate procedures to multivariate procedures (Worsley et al. 1997) reflects this natural progression of characterizing functional architectures in the brain. So, why are hierarchies so important? A hierarchy is defined in terms of a distinction between forward and backward connections (Felleman and Van Essen 1991). This corresponds to an asymmetry in recurrent effective connectivity. This meant that to create a generative model of cortical hierarchies, it was necessary to introduce dynamic causal modeling as a complement to the study of correlations (ie functional connectivity). This follows from the fact that the correlation between two brain areas is the same in both directions, which is clearly a poor metric of functional integration in the hierarchical brain. In one sense, we also return to the underlying role of generative models—not of brain imaging data—but of the computational anatomy of the brain itself (Friston et al. 2017b). This led to an engagement of the extended SPM software with computational neuroscience and the modeling of choice behavior that could be integrated in applications such as computational fMRI (Friston and Dolan 2010), detailed in the *Behavioral modeling* section above.

At the final session of the most recent SPM short-course in London, we were asked about the future of SPM. Our answer, of course, was to pursue the eternal task of developing the right kind of generative models—and their inversion schemes—that enable people to answer their questions. This will depend upon the questions posed by the community, as it joins the dots between systems neuroscience, functional genomics, cell biology, and social neuroscience. In this sense, the agenda of SPM is just to socialize evidence-based neuroscience.

## Footnotes

1 More specifically, DCM yields an approximation of the log-evidence called the Evidence Lower Bound (ELBO) in machine learning or the *negative variational free energy* in statistical physics. It is usually referred to as the *free energy* in neuroimaging.
